# Liver Transplantation Using an Iliac Artery Graft for a Patient With a Celiac Artery Aneurysm: A Case Report

**DOI:** 10.7759/cureus.86096

**Published:** 2025-06-15

**Authors:** Jiro Kimura, Prakash Chauhan, Matthew Cooper, Calvin Eriksen, Raj Prasad

**Affiliations:** 1 Transplant Surgery, Medical College of Wisconsin, Milwaukee, USA

**Keywords:** celiac artery aneurysm, iliac artery conduit, liver cirrhosis, liver transplantation, supraceliac aorta

## Abstract

Celiac artery aneurysm is a rare entity among the visceral artery aneurysms, with a high risk of rupture, resulting in high mortality. Arterial complications, such as thrombosis, dissection, and hemorrhage, are among the most serious complications after liver transplantation (LT), which can lead to abscess formation, ischemic cholangiopathy, and hepatic ischemia and necrosis. Therefore, an adequate inflow of the hepatic artery is crucial to avoid any occlusion after LT. When a normal arterial anastomosis is not appropriate, the use of an artery conduit is an important option for graft vascularization.

Herein, we present a patient who underwent LT using an iliac artery conduit due to a celiac artery aneurysm. A 53-year-old man with a history of alcoholic cirrhosis presented to the Emergency Department of our institution with abdominal pain and jaundice. He was diagnosed with alcoholic cirrhosis and admitted to our hospital the same day. A preoperative contrast-enhanced computed tomography (CT) scan revealed a celiac artery aneurysm with dissection. Therefore, hepatic artery reconstruction with an iliac artery conduit was planned. Twenty days after the initial visit, he underwent transplantation. At the time of surgery, the supraceliac aorta was exposed after his native liver was explanted. After cavo-cavostomy and portal vein anastomosis were performed, the iliac artery conduit was anastomosed with the supraceliac aorta and the implanted common hepatic artery, with a running suture. On postoperative day 2, the patency of the iliac artery conduit was confirmed by contrast-enhanced CT scan. His postoperative course was uneventful, without any vascular complications. For patients with a celiac artery aneurysm, the use of an iliac artery conduit in LT can be performed safely. This approach may become a promising alternative for future treatment.

## Introduction

Celiac artery aneurysm is a rare entity among the visceral artery aneurysms. Etiology of this entity includes atherosclerosis, infection, and congenital, developmental, and inflammatory conditions [1]. Rupture of the aneurysm results in high mortality (13%-50%) [2]. These aneurysms usually require early detection and prompt treatment to reduce mortality [3].

Patients with a celiac artery aneurysm pose technical challenges for liver transplantation (LT). Under normal anatomical conditions, without a celiac artery aneurysm, end-to-end anastomosis of the donor and the recipient common or proper hepatic artery is performed. When a normal arterial anastomosis is not appropriate, the use of an artery conduit is an important option for graft vascularization [4-7]. These artery conduits are usually implanted in the abdominal aorta of the recipient in LT.

To the best of our knowledge, LT using an iliac artery conduit in the presence of a celiac artery aneurysm associated with median arcuate ligament syndrome (MALS) has not been reported yet. Herein, we present a patient who underwent LT using an iliac artery conduit due to a celiac artery aneurysm. 

## Case presentation

**Figure 1:**
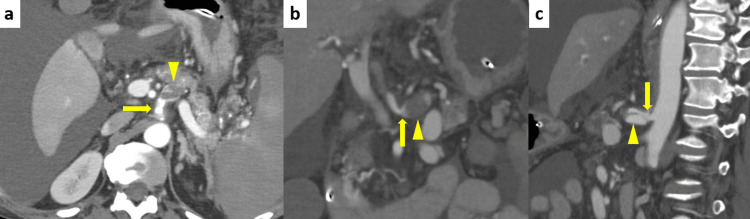
Contrast-enhanced abdominal computed tomography scan (a) Axial view: The 23-mm aneurysm with dissection and thrombus (arrowhead) was located at the celiac artery (arrow). (b) Coronal view and (c) Sagittal view: Compression of the celiac artery origin by the median arcuate ligament (arrow), and post-stenotic dilation (arrowhead), are shown.

A 53-year-old man with a history of alcoholic cirrhosis presented to the Emergency Department of our institution with constant and diffuse abdominal pain and jaundice. On physical examination, his abdomen was distended but not tender. A computed tomography (CT) scan revealed massive ascites and a cirrhotic liver. His bilirubin, aspartate aminotransferase (AST), and alanine aminotransferase (ALT) levels were elevated to 31.6 mg/dL, 1543 IU/L, and 464 IU/L, respectively. He was diagnosed with decompensated liver cirrhosis (Child-Pugh score 14 and Class C) and Grade 3 acute-on-chronic liver failure, and was admitted to the hospital on the same day. After admission, he was managed medically while awaiting LT. A preoperative contrast-enhanced CT scan revealed a 23-mm aneurysm with dissection and thrombus at the celiac artery (Figure 1). It was assumed that the etiology was MALS. Therefore, hepatic artery reconstruction with an iliac artery conduit on the supraceliac aorta was planned, because the infrarenal aorta was calcified. His Model for End-Stage Liver Disease (MELD) score was 43 prior to surgery.

**Figure 2:**
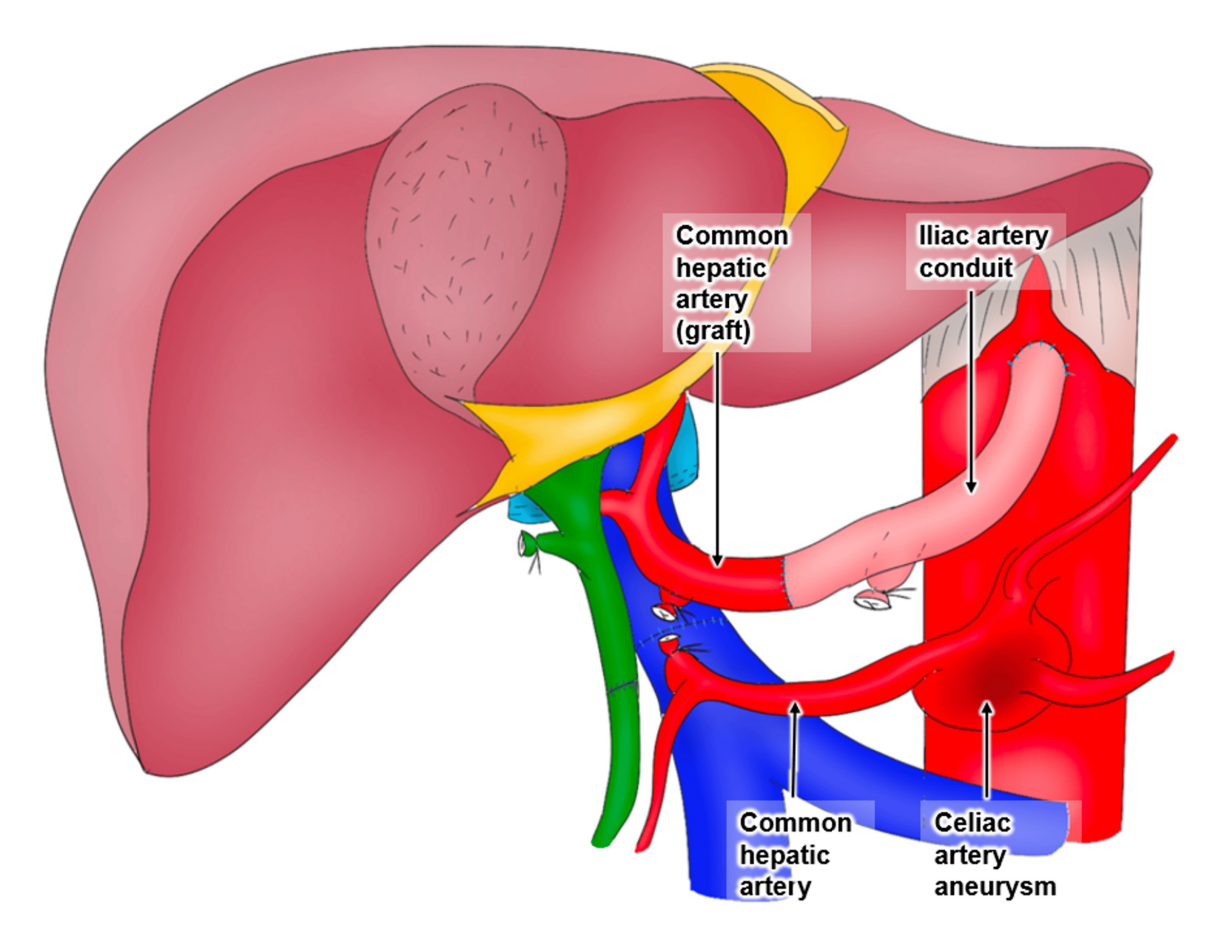
Operative findings The artery was reconstructed using the iliac artery conduit between the supraceliac aorta and the common hepatic artery of the graft. Image credit: Jiro Kimura

**Figure 3:**
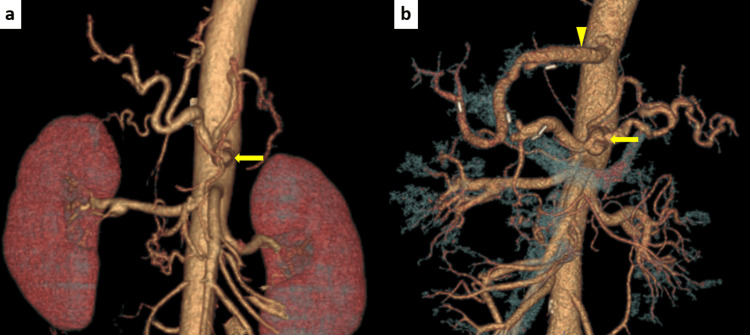
3D-computed tomography angiogram (a) Before liver transplant and (b) After transplant The iliac artery conduit was patent (arrowhead), and the size of the celiac artery aneurysm was not changed (arrow).

Twenty days after his initial visit, he underwent transplantation. After removing his native liver, the diaphragmatic crura were opened to expose the supraceliac aorta. The supraceliac aorta was dilated, with a thin wall. A small Satinsky clamp was placed in a side-biting manner. An arteriotomy was made with a blade. The previously prepared donor iliac artery conduit was sewn end-to-side to the aorta using 5-0 prolene running sutures. After performing side-to-side cavo-cavostomy and portal vein anastomosis, the peripheral side of the iliac artery conduit was anastomosed to the implanted common hepatic artery using a running suture of 5-0 prolene (Figure 2). Finally, the choledochocholedochostomy was performed. Control of anastomotic bleeding was difficult due to the thin aortic wall and abnormal coagulation. His abdomen was packed with gauze that day. On postoperative day 2, the patency of the iliac artery conduit was confirmed by a contrast-enhanced CT scan (Figure 3). On day 3, the gauze packing was removed, and the choledochocholedochostomy was revised due to bile leakage. 

Following the second operation, the patient was discharged home on postoperative day 61 without any vascular complications, rejection, or severe infection, although his postoperative course was complicated by ileus and malnutrition, and he required rehabilitation. A contrast-enhanced CT scan three months after surgery revealed no vascular complications, such as stenosis, dissection, or thrombosis. During the seven-month follow-up period, he did not experience any vascular complications, including rupture of the celiac artery aneurysm. 

## Discussion

To the best of our knowledge, this was the first case report of LT using the iliac artery conduit for the celiac artery aneurysm. Interestingly, it was markedly effective in the present patient, a 53-year-old Caucasian male.

The median arcuate ligament is a fibrous arch that joins the left and right crura of the diaphragm on either side of the aortic hiatus, crossing the aorta superior to the celiac artery origin. When this anatomy is associated with symptoms such as epigastric pain or vomiting, it is named MALS [8]. MALS can occur when the median arcuate ligament has a lower insertion and compresses the proximal portion of the celiac artery, potentially leading to distal splanchnic artery aneurysms due to hemodynamic alteration. We have shown that LT using the iliac artery conduit, bypassing the celiac artery aneurysm caused by MALS, can be performed safely without arterial complications. A major concern with leaving the celiac artery aneurysm is the risk of later rupture. Once rupture occurs, it becomes life-threatening, and mortality is high [9]. Previous studies have reported that the incidence of rupture ranges from 5% (<2.0 cm) to 60% (>3.2 cm) [10]. Although there are no absolute size criteria to guide treatment decisions, lesions >2 cm are generally considered to require treatment [11]. However, considering our patient's high MELD score of 43, the etiology of MALS, and the invasiveness of LT, revision of the celiac artery aneurysm should not be considered. In addition, in the case of resection and arterial anastomosis for the celiac artery, there is always the risk of bleeding or thrombosis. Therefore, our policy was reasonable. In future follow-up, if the aneurysm were to enlarge, therapeutic intervention - such as coil embolization via interventional radiology - could be considered [12]. 

In the selection of the arterial reconstruction method, there were several options: a supraceliac iliac artery graft, an infrarenal iliac artery graft, and a gastroduodenal artery (GDA) inflow derived from the superior mesenteric artery with ligation of the common hepatic artery. In fact, there is some risk of complications when choosing the supraceliac approach. These include possible early hepatic artery occlusion and inferior graft survival rates compared with non-conduit techniques [12,13]. However, there were specific reasons to choose the supraceliac artery approach. First, calcifications were seen at the infrarenal aorta in this patient. Second, the GDA was congenitally small; therefore, blood inflow to the liver graft was not guaranteed. Thus, the supraceliac approach was the best option for this patient.

The present study demonstrates the feasibility and utility of the iliac artery conduit for LT in patients with a celiac artery aneurysm. However, the long-term outcomes in these patients remain unclear. Therefore, further collection of cases is necessary.

## Conclusions

This case highlights the feasibility and safety of using an iliac artery conduit for hepatic artery reconstruction during LT in the presence of a celiac artery aneurysm. Despite the technical complexity, the supraceliac aorta proved to be a suitable inflow source, avoiding the risks associated with aneurysm manipulation or alternative arterial reconstructions. The patient experienced no vascular complications postoperatively, supporting the viability of this approach in select cases. While long-term outcomes remain to be clarified, this case may serve as a valuable reference for managing similar vascular challenges in LT.
